# Chitosan/Gelatin/PVA Scaffolds for Beta Pancreatic Cell Culture

**DOI:** 10.3390/polym13142372

**Published:** 2021-07-20

**Authors:** Yesenia Sánchez-Cardona, Claudia E. Echeverri-Cuartas, Marta E. Londoño López, Natalia Moreno-Castellanos

**Affiliations:** 1 Grupo de Investigación en Ingeniería Biomédica EIA (GIBEC), Programa de Ingeniería Biomédica, Escuela de Ciencias de la Vida, Universidad EIA, km 2 + 200 Vía al Aeropuerto José María Córdova, Envigado 055428, Colombia; yesenia.sanchez@eia.edu.co (Y.S.-C.); claudia.echeverri@eia.edu.co (C.E.E.-C.); marta.londono@eia.edu.co (M.E.L.L.); 2 CINTROP, Department of Basic Sciences, Medicine School, Health Faculty, Universidad Industrial de Santander, Cra 27 calle 9, Bucaramanga 680002, Colombia

**Keywords:** scaffold, biomaterials, three-dimensional culture, ternary blend scaffolds, medicine regenerative, cytotoxicity

## Abstract

Chitosan scaffolds based on blending polymers are a common strategy used in tissue engineering. The objective of this study was evaluation the properties of scaffolds based on a ternary blend of chitosan (Chi), gelatin (Ge), and polyvinyl alcohol (PVA) (Chi/Ge/PVA), which were prepared by cycles of freeze-thawing and freeze-drying. It then was used for three-dimensional BRIN-BD11 beta-cells culturing. Weight ratios of Chi/Ge/PVA (1:1:1, 2:2:1, 2:3:1, and 3:2:1) were proposed and porosity, pore size, degradation, swelling rate, compressive strength, and cell viability analyzed. All ternary blend scaffolds structures are highly porous (with a porosity higher than 80%) and interconnected. The pore size distribution varied from 0.6 to 265 μm. Ternary blends scaffolds had controllable degradation rates compared to binary blend scaffolds, and an improved swelling capacity of the samples with increasing chitosan concentration was found. An increase in Young’s modulus and compressive strength was observed with increasing gelatin concentration. The highest compressive strength reached 101.6 Pa. The MTT assay showed that the ternary blends scaffolds P3 and P4 supported cell viability better than the binary blend scaffold. Therefore, these results illustrated that ternary blends scaffolds P3 and P4 could provide a better environment for BRIN-BD11 cell proliferation.

## 1. Introduction

To mimic in vivo microenvironments is essential to maintain survival and to understand cell biology. The three-dimensional (3D) culture has been increasingly used for this purpose. The 2D culture does not represent the physiology of the native environment of the cells, and neither does it favor cell survival due to the lack of cell- extracellular matrix (ECM) interaction. As a consequence, the phenotype of cells is affected, leading to necrotic death and apoptopic cells. For this reason, scaffolds have been explored as 3D supports in cell culture, implementing biomaterials with physical, chemical, and biological characteristics, giving mechanical support, and getting more closer to the native morphology [1,2,3,4,5]. Importantly, cells in 3D culture have better survival, vascularization, gene and hormone expression; cell interactions, cell proliferation and differentiation, etc. [1,3,4,5,6,7,8].

For this reason, different types of biomaterials of natural or synthetic origin are being implemented to make scaffolds in order to increase the replication of the pre-existing beta cells or improve their function in vivo and in vitro [9]. However, most of these scaffolds do not mimic the complexity of the composition and structure of the pancreatic ECM. They do not favor focal adhesions and cell-cell or cell-material interactions. Therefore, it is still a complex problem to keep these cells in culture due to their complex regulatory mechanisms, dependence on oxygen, the architecture of their native environment, the transport of nutrients, and the low proliferation rate in vivo and in vitro [10].

The islets of Langerhans are surrounded by a membrane of the ECM. This ECM, in turn, is composed of laminin, different types of collagens, and fibronectin, which binds to the integrins on the surface of the islet to provide structural support. This binding mediates cell adhesion by activating intracellular signaling pathways [11]. In addition, ECM serves as a reservoir of growth factors which are released to regulate the behavior of proximal cells, suppression of apoptosis, proliferation, and cell migration [12,13]. It has been shown that the use of some biomaterials to mimic the ECM of pancreatic islets is feasible [14] and that a 3D architecture is necessary to compensate for the absence of peripheral basement membranes to avoid progressive loss and islet functionality [15]. Some of these biomaterials are capable of providing structural and mechanical support through scaffolds for cells to adhere, allowing the pass of nutrients and oxygen [16], being able to provide an environment for cells to receive biochemical stimuli, which allows cell differentiation, migration and proliferation [17,18].

To cultivate or encapsulate β or pancreatic islet cells, various types of natural and synthetic materials have been used, such as: poly (ether sulfone)/polyvinylpyrrolidone (PES/PVP) [16], poly (lactide-glycolide) (PLG) [19], polyglycolic acid (PGA) [20], poly-p- dioxanone (PDS) [18], poly (ethylene oxide terephthalate)/poly (butylene terephthalate) (PEOT/PBT) [21], (poly-L-lactic acid) (PLLA), poly (dimethyl)siloxane (PDMS), poly (D,L-lactic acid) (PDLLA) [22], silk fibroin (SF)/(P-dECM) which is derived from pig pancreas after decellularization [23], polylactic-co-glycolic acid (PLGA) [24], PLGA/collagen [25], polyethylene glycol (PEG), alginate, collage, polytetrafluoroethylene (PTFE), polydimethylsiloxane (PDMS), poly (ethylene-vinyl acetate) (PEVA), polytetrafluoroethylene (PTFE), polydimethylsiloxane (PDMS), polycaprolactone (PCL) [17], chitosan, collagen, alginate, PGA, polylactic acid (PLA) [26], dextran, gelatin [1], hyaluronic acid [27], PLGA/ poly (lactide-co- glycolide) (PLG) [15]. Most of these studies focus on a single material or binary mixtures, and most scaffolds had to be modified or supplemented with ECM components and decellularized tissues. The scaffolds designed from a single biomaterial of natural origin were porous and interconnected but fragile and rapidly degrading, so they did not allow revascularization of the islets. The synthetic origin ones exhibited more stable structural support and slower degradation due to their good mechanical properties, and reduced viability and functionality of islet cells due to polymer biodegradation and limited permeability. Ternary and binary blend scaffolds were implemented to improve degradation and mechanical properties. Nonetheless, although each biomaterial has different properties that make them attractive for β cell culture, all of these properties do not necessarily add up when mixed [28].

Chitosan (Chi) is a natural polymer that is obtained from the partial deacetylation of chitin, being one of the most abundant aminopolysaccharides in nature since it is found in the exoskeletons of crustaceans such as shrimp, crabs, etc. the skin of tilapia and the cell walls of some fungi [29]. This material has bioactive, hemostatic, microbial activity, biocompatibility, physicochemical properties, and biological activity [30,31]. Gelatin (Ge) is obtained from the partial hydrolysis of collagen. This, like chitosan, is a biodegradable and biocompatible material, in addition to having other properties such as low immunogenicity. Furthermore, gelatin is made up of various amino acids, and this composition could contain the RGD tripeptide sequence (arginine/glycine/aspartic acid). These, in turn, are found in ECM, present in adhesion proteins such as fibronectin, which, when interacting with the integrins of the cell membrane, promote cell adhesion, triggering the biological response. Therefore, the addition of gelatin can favor the adhesion, proliferation, and differentiation of beta cells in the culture medium [32]. This biomaterial has been implemented in various studies as a support for other materials such as chitosan, where it has been shown that gelatin helps increase porosity and provides greater rigidity to scaffolds [33]. Finally, polyvinyl alcohol (PVA) is obtained by saponification of poly (vinyl ester) or (vinyl ether). It has characteristics such as its excellent chemical resistance, physical properties, high resistance to traction and compression, non-toxicity, and biocompatibility. Therefore, it will not cause any toxicity or stimulation to beta cells [34,35].

This study aims to develop Chi/Ge/PVA scaffolds with different weight ratios for culture beta cells. The advantages of the over previously mentioned scaffold are that it: (1) promotes the attachment, adhesion and spread of beta cells; (2) it is mechanically stable to protect against physical stresses, being able to support and transfer loads correctly.; and (3) it is highly porous, interconnected and with various types and sizes of pores that promote fast vascular ingrowth. Polymer scaffolds porous were prepared by freeze-thawing cycles and lyophilization. Scaffolds were characterized for their water uptake, mechanical properties, porosity, and controllable degradation rate.

## 2. Materials and Methods

### 2.1. Materials 

Polyvinyl alcohol (PVA) (MW: 89,000–98,000, degree of hydrolysis: 99+%), chitosan (Chi) (228.339 Da, medium molecular weight, 86.36% degree of deacetylation), and gelatin (Ge) from bovine skin type B were purchased from Sigma Aldrich (St. Louis, MO, USA). Acetic acid was purchased from Chemí (RA Chemicals, ABD laboratorios, Bógota, Colombia). Other reagents used in this investigation were of analytical grade. Chitosan was further purified. Ultrapure water was also used during all procedures.

### 2.2. Preparation of Chitosan, Gelatin, and PVA Solution 

Chitosan was dissolved in 1% *v*/*v* CH_3_COOH solution at a concentration of 2% *w*/*v* under magnetic stirring at 450 rpm for 12 h at 37 °C. PVA polymer was dissolved in the ultrapure water at 85 °C to obtain a solution of 2% *w*/*v* under magnetic stirring at 450 rpm for 4 h. A 2% *w*/*v* gelatin solution was prepared by adding gelatin in the ultrapure water, under magnetic stirring at 450 rpm for 4 h at 40 °C.

### 2.3. Preparation of Samples

**Figure 1:**
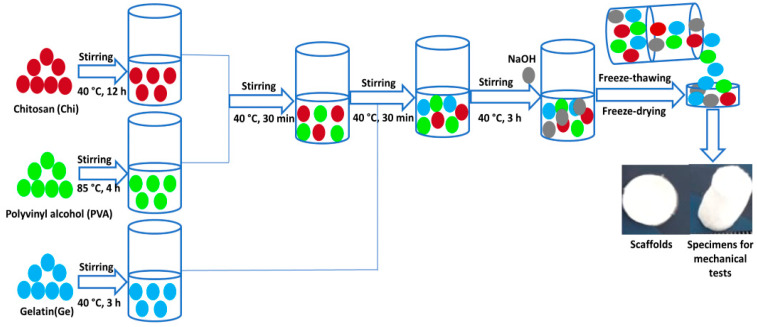
Schematic illustration of the preparation process of the sample’s scaffolds.

The preparation process for the samples is shown in Figure 1 with the weight ratio of Chi and PVA, as indicated in Table 1. Chi/PVA was blended at 40 °C and stirred at 800 rpm for 30 min until a homogenous blend. After this time, the Gelatin solution was added to the Chi/PVA solution in the weight ratio indicated in Table 1. All prepared solutions were mixed with ratios of 1:1:1, 2:2:1, 2:3:1, and 3:2:1 (*w*/*w*) of chitosan, gelatin, and PVA, solution respectively, under magnetic stirring at 800 rpm for 30 min at 40 °C to form a homogeneous solution. The polymer solutions pH was adjusted with sodium hydroxide solution (5 M) at pH 5.5 (below the pKa of the primary amine of chitosan (~6.5) and the isoelectric point of gelatin) to obtain a homogeneous polymer blends solution [36,37]. After adjusting the pH, the solutions were left under magnetic stirring for a further 3 h more. Finally, the solution was filtered, and from it, aliquots of 7 mL were poured into glass Petri dishes. Additionally, 450 µL were poured into cylindrical molds, and freeze-thawing nine cycles were performed at −50 °C for 8 h and 25 °C for 8 h. Finally, the scaffolds were freeze-dried using an SP VirTis AdVantage Pro Freeze Dryer (SP Industries, Inc., Warminster, UK).

### 2.4. Morphological Properties

Morphological observation of the surface, and cross-section of the scaffolds were performed by scanning electron microscopy (SEM), using a Phenom Pro X (PhenomWorld, Phenom ProX, Eindhoven, The Netherlands). SEM images were acquired with an accelerating voltage of 15 kV and magnifications of 500×. The morphology and microstructure of the scaffolds was characterized. Moreover, analysis and processing of SEM images were performed by MATLAB^®^ software (MathWorks, Natick, MA, USA).

### 2.5. Porosity Measurement

The density and porosity values of the scaffolds were determined by liquid displacement, using a method proposed by Zhang and Peter [38]. In this method, distilled water was used as the displacement liquid. A scaffold sample of weight (W_s_) was immersed in a graduated cylinder containing a known volume (V_1_) of water. The sample was kept in the liquid for 48 h. Afterward, the total volume of liquid and the distilled water impregnated scaffold was recorded as V_2_. The volume difference (V_2_ − V_1_) was the volume of the skeleton of the scaffold. The liquid impregnated scaffold was removed from the cylinder, and the residual liquid volume was recorded as V_3_. The volume of the liquid held in the scaffold was determined as (V_1_ − V_3_). Thus, the total volume of the scaffold was (Equation (1)):

(1) $$V=\left(V_{2}-V_{1}\right)+\left(V_{1}-V_{3}\right)=V_{2}-V_{3}$$

The density of the scaffold (⍴) was expressed as (Equation (2)):

(2) $$⍴=\frac{W_{s}}{\left(V_{2}-V_{3}\right)}$$

and the porosity percentage of the scaffold (%φ) was obtained by (Equation (3)):

(3) $$\%\varphi =\frac{\left(V_{1}-V_{3}\right)}{V}$$

### 2.6. Infrared Spectroscopy

Structural properties in the scaffolds were characterized by Fourier Transform Infrared—Attenuated Total Reflectance (FTIR-ATR), using a Spectrum One spectrometer (Perkin-Elmer, Shelton, CT, USA). All spectra were taken in the spectral range of 4000–500 cm^−1^ with a resolution of 4.0 cm^−1^ and 32 scans. The spectrums obtained got normalized with an ordinate limit of up to 1.0 of absorbance using the tool available in the spectrometer’s software (SPECTRUM, v.10.5.3, Perkin-Elmer Inc., 2016, Shelton, CT, USA).

### 2.7. Swelling Test

The swelling percentages of the scaffolds were measured using the gravimetric method. The swelling percentages were evaluated by immersing pre-weighed, in an analytical balance (ES 1255M, Precisa Gravimetrics, Dietikon, Switzerland), dry scaffold sample ($W_{d}$) of 10 × 10 mm into 4 mL of phosphate-buffered saline solution (PBS, pH 7.4) and incubated at 37 °C, in a vacuum oven (Lindberg/Blue M, Thermo Fisher Scientific, MA, USA), for 3, 7, 14, 21, and 28 days, the buffer was replaced every three days. At certain intervals, the samples were taken out and put on a filter paper to remove the excessive PBS and the weight gain of the swollen film (W_s_). This process was continued until reaching the constant weight in three repeating measurements. The swelling percentage (SP) was calculated according to Equation (4) [39]:

(4) $$\mathrm{SP}\%=\frac{W_{s}-W_{d}}{W_{d}}\times 100$$

where W_s_ is the weight of swollen scaffolds samples (mg), and W_d_ is the weight of dry scaffolds samples (mg). The measurements were repeated three times for each type of scaffold. 

### 2.8. Degradation Test

The degradation of the rectangular samples (10 × 10 mm) was carried out in a phosphate buffer solution (PBS) of pH = 7.40 at 37 °C for 28 days. The buffer solution was replaced 3–4 every day. The samples were immersed in vials containing 4 mL of PBS. The degradation process was monitored by determining water absorption and loss of material mass. The samples were weighed on day 3, 7, 14, 21 and 28. The percentage degradation (DP%) was expressed as degradation in percentage (%) of original weight samples using equation Equation (5) [39]:

(5) $$\mathrm{DP}\%=\frac{W_{d}-W_{t}}{W_{d}}\times 100\;$$

where W_t_ is the final weight scaffolds samples (mg), and W_d_ is the weight of dry scaffolds samples (mg). The measurements were repeated three times for each type of scaffold.

### 2.9. Design, Prototyping, and Manufacture of Molds

**Figure 2:**
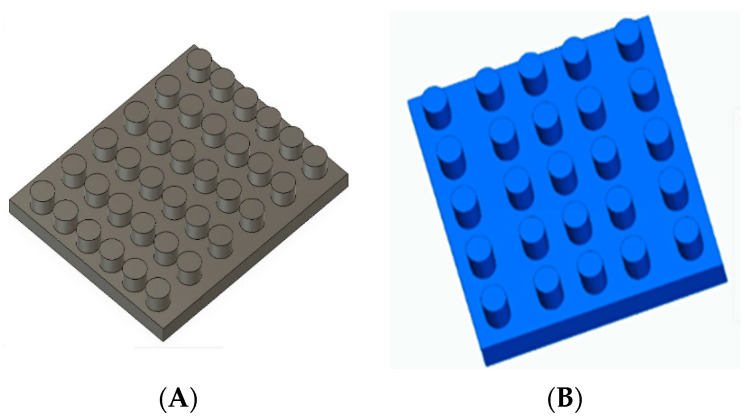
3D designs for scaffolding. (**A**) molds for scaffolds implemented in cell culture and (**B**) molds for test tubes to evaluate mechanical properties.

Two molds were designed in the Solid Edge^®^ software (Siemens, Plano, TX, USA), as shown in Figure 2, to have greater control over the geometry and size of the scaffolds. For circular scaffolds were designed a mold (A) of approximately 11 mm in diameter and 5 mm in height; and a second mold (B) for the specimens that would be used for mechanical tests of 9 mm in diameter and 15 mm in height. After the CAD design, the prototypes were printed on a 3D printer (MakerBot Replicator 2X, 3D Solutions S.A.S, Bógota, Colombia), and finally, the molds were manufactured in silicone rubber material, leaving the bottom of each glass mold.

### 2.10. Compressive Mechanical Properties

**Figure 3:**
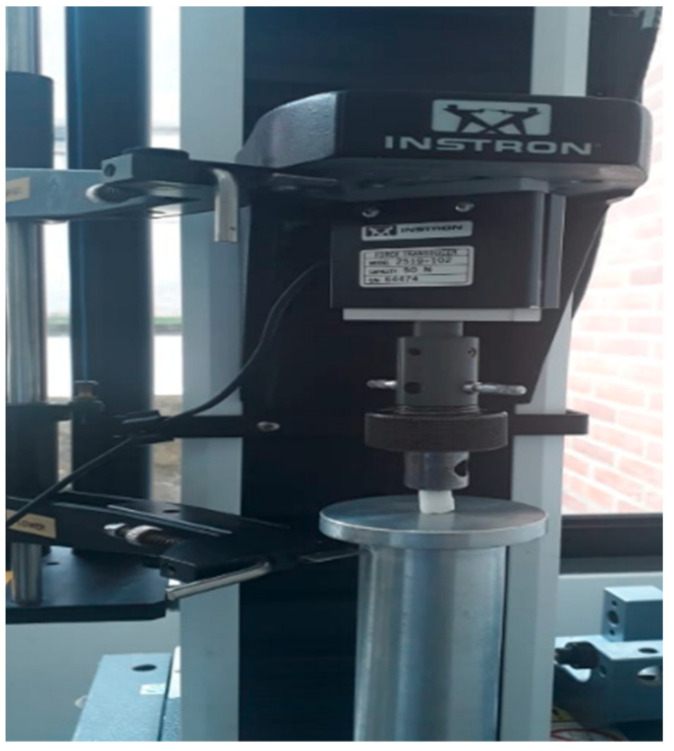
Assembly illustration for mechanical testing.

The mechanical properties of the scaffolds were measured in unconfined uniaxial compression, as shown in Figure 3. All samples had a cylindrical shape and were approximately 13–15 mm in height (*n* = 8). The test was performed by a universal testing machine (Instron, model 3345), according to standard method ASTM D 882-09 [40]. Scaffolds were loaded with a 50 ± 0.25 N load cell at a displacement rate of 1 mm/min until deformation of 50% of its initial length. Without preload (in preliminary tests, the sample starts at zero stresses and zero deformations) and operating at room temperature. The compressive stress of the scaffolds was determined as indicated in the equation Equations (6) and (7) [41]:

(6) $$E=\frac{\sigma }{\varepsilon }$$

where σ is the stress measured in the elastic range of the stress versus strain curve and ε the strain obtained in the elastic range, calculated as:

(7) $$\varepsilon =\frac{L_{f}-L_{i}}{L_{i}}$$

where L_f_ is the final length of the specimen and L_i_ its initial length.

### 2.11. Cell Culture

BRIN-BD11 cells (Cell Bank, Coleraine, Northern Ireland, U.K) derived from rat pancreatic islets 4 × 10^4^ cells/cm^2^ were grown in DMEM-F12 -GlutaMAX-I (Gibco, New York, NY, USA) medium containing 10% heat-inactivated fetal calf serum, penicillin (100 U/mL), streptomycin (100 μg/mL). Cultures were maintained at 37 °C under 5% CO_2_ and in a humidified atmosphere. Subcultures were established once every 3–4 days by trypsin/EDTA treatment.

### 2.12. MTT Assay

The metabolic activity of cells after 48 h of treatment was determined using the 3-(4,5-dimethylthiazol-2-yl)-2,5-diphenyltetrazolium bromide (MTT, Sigma Aldrich, St. Louis, MI, USA) assay, as described before [42] and by the manufacturer. Briefly, after the addition of the MTT reagent to the samples, the purple formazan crystals formed were dissolved in DMSO (99.7%) (Sigma Aldrich, St. Louis, MO, USA), and the absorbance was read at 570 nm on a Synergy H1 microplate reader (Biotek, Winooski, VT, USA). DMEM F12 medium was used as a negative control, and Triton X-100 (1%) was used as a positive control.

### 2.13. Statistical Analysis

Triplicate experiments were performed for each sample, the results were presented as mean ± SD, and the *p*-value was calculated using an independent *t*-test was carried out to determine the statistical significance (*p* < 0.05) using MINITAB (version 19.2; Minitab, Inc., Coventry, UK) to find differences between scaffolds in terms of their porosity, pore size distribution, mechanical property, swelling and degradation. The statistically significant differences among different variables were performed using a one-way analysis of variance (ANOVA). If treatments were determined to be significant, pairwise comparisons were performed using Tukey, Games-Howell, or Bonferroni’s adjustment, and differences were considered significant for *p*-values < 0.05. Additionally, Pearson’s correlation was performed. Finally, in some cases, multivariate analyzes were performed.

## 3. Results and Discussion

### 3.1. Microstructure

**Figure 4:**
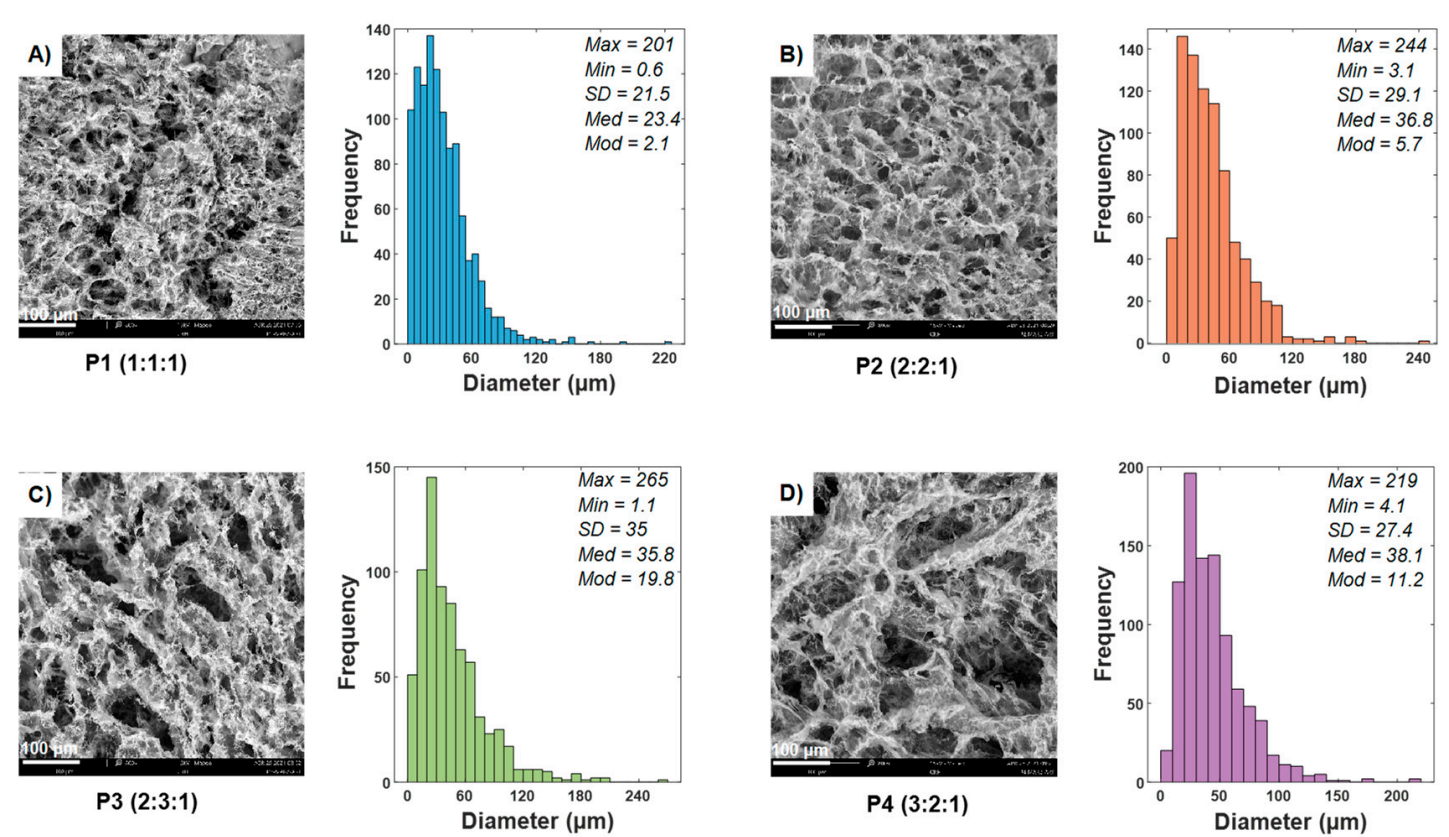
SEM images of ternary blend scaffolds (**A**) 1:1:1, (**B**) 2:2:1, (**C**) 2:3:1 and (**D**) 3:2:1. The scale bars are shown in the micrographs.

SEM micrographs at 500× magnification and pore diameter, median, mode, standard deviation, and morphological characteristics of the ternary blends are presented in Figure 4. Ternary blend scaffolds P3 and P4 have larger pores compared to the P1 and P2 samples. Ternary blend scaffolds show significant differences compared to controls (*p* < 0.05). Multiple comparisons were made to see significant differences between a pair of groups (*p* < 0.05). The P1 ternary blend scaffolds showed significant differences compared to all the other samples, except for sample P7. The P2 ternary blend scaffolds showed significant differences compared to the samples P1, P5, P8, P7, P9, and P10. The frameworks of ternary blend P3 showed significant differences with respect to P1 and all the controls. Finally, the P4 ternary blend scaffolds presented significant differences with respect to P1, P5, P7, P8, P9, and P10.

**Figure 5:**
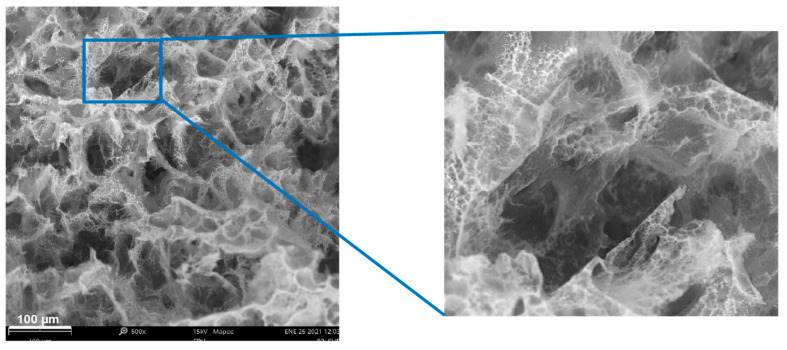
Ternary blend scaffold P3 at the scale bars is shown in the micrograph, interconnected walls, and micropores inside the largest pores.

In general, it was found all scaffolds have a three-dimensional network structure. In addition, the microstructure of the scaffolds has a lot of micropores, and these pores are uniformly distributed on the surface, as shown Figure 5. These results agree with those obtained by Fan et al., 2016 [35]. The ternary blend scaffolds presented a mean pore opening of 28 to 46 µm and pore diameter sizes between 0.6–265 µm, and all the scaffolds showed open, blind, and closed pores. 

All controls had a maximum pore diameter size < 180 µm except the chitosan control scaffolds, which had pore sizes similar to the ternary blend scaffolds. The controls had a mean aperture of 16–102 µm, minor than ternary blend scaffolds and less frequently. The PVA scaffold presented the most compacted structure with dense walls and very few pores, so it could be observed that this scaffold had no interconnectivity (data not shown), which can affect the cell viability.

The scaffolds porosity plays an essential role for the cells since the porous structures allow the growth of blood vessels and peripheral nerves and the deposition of ECM, cell growth, and favors the transport of gases and nutrients through the polymeric network. Also, scaffolds must have several types of pores since each one contributes a favorable characteristic in cell culture; open pores are essential for cells to penetrate the structure [43]. These pores are important for keeping cells alive in culture, as they allow the transport of nutrients, oxygen, and waste products into the scaffold. Closed pores give structural stability to the scaffold, and blind pores can reduce the diffusion distance of potential degradation products and soluble gases [16,44].

As the same that porosity, the pores size, and their interconnectivity is also essential, several studies have also shown that nanopores (<2 nm) promote cell adhesion and reabsorption at controllable rates as well as the mesopores 2–50 nm. In addition, these mesopores are necessary for the release of insulin since it ranges from 1.35 to 2.75 nm, as it does the stimulation of glucose from the cells since the glucose is approximately 0.4 nm [16,45]. Macroporous in the range of 30–40 µm maximizes vascularization [16], while macroporous in the range of 100–200 µm promotes cell proliferation, helping to revascularize and transport gases nutrients to the outside of the scaffold [4,45,46,47]. Finally, macroporous in the range from 250 to 425 µm increase the expression of insulin and key markers in the modulation of β cells and promote rapid migration of cells into the scaffold, which induces regeneration in vivo [6,46].

### 3.2. Porosity Measurement

Table 2 shows the significant differences (*p* < 0.05) in the percentage of porosity of the samples. We can also observe that the ternary blend scaffolds have a higher percentage of porosity than some controls. The porosity of ternary blend scaffolds was higher at 80%, while the porosity of single polymer scaffolds varies between 55.56 ± 9.62% and 90.56 ± 1.53%. An independent t-test was used to find *p*-value and statistical significance. Multiple Tukey comparisons were performed to see significant differences between a pair of groups (*p* < 0.05). Significant differences were found in the percentage of porosity in a pair of groups: P2-P10, P4-P10, P1-P5, P2-P7, P3-P7, and P4-P7.

Porosity is one of the most important parameters in scaffolds since it is directly related to the volume/surface ratio. This parameter is fundamental because it can affect cells adhesion to the scaffold. Furthermore, porosity influences the transport of oxygen, carbon dioxide, and nutrients which are essential to keep cells alive. Finally, porosity also influences the swelling capacity, the percentage of degradation, mechanical stability, and anisotropy; the latter can affect or favor the migration, viability, and alignment of cells [43].

### 3.3. FTIR Spectra of Samples

**Figure 6:**
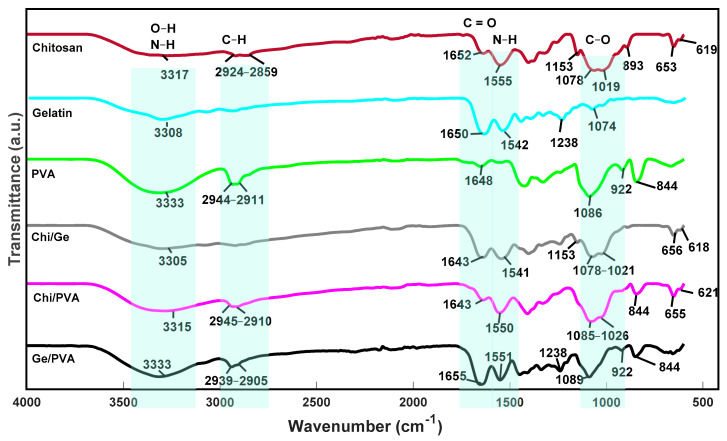
FTIR spectra of scaffolds control.

In Table 2, we observe the labels and color codes of all scaffolds for better compression. The chemical structure of the scaffolds was analyzed using FT-IR spectroscopy. The spectra of the pure polymer scaffolds and the possible mixtures between them are shown in Figure 6. The spectrum of the chitosan scaffold exhibited the characteristic bands at 1153 cm^−1^ assigned to the C–O stretching and the broadband above 3000 cm^−1^ correspondence to the stretching of the O–H and N–H bonds [48,49,50,51]; the band at about 2924–2859 cm^−1^ stretch of C–H and that at 1652 cm^−1^ attributed to the stretching of C=O of the amide group, the vibrational band at about 1555 cm^− 1^ assigned to the bending of N–H and that at 1378 cm^−1^ stretching of the C–N group and symmetric bending of the CH_3_ bond [52,53]. The characteristic bands of the spectrum of the gelatin scaffold were detected, which occur at 3308 cm^−1^ due to amide A that represents the N–H vibration. The vibration bands at 1238 cm^−1^ attributed to the N–H group (flexion), and that at 1650 cm^−1^ the stretching of the C=O group of Amide I. The band at around 1542 cm^−1^ flexion of N–H of the Amide II group and tension of the C–N group [51,54,55]. Finally, the FT-IR spectra for the PVA hydrogels presented the characteristic bands at 3333 cm^−1^ correspondings to the linked H atoms, intense and well defined, indicating the presence of water in the scaffold; furthermore, it suggests the stretching of hydrogen bonds in the polymeric chains of the scaffold [56]. The vibrational band at about 2944 and 2911 cm^−1^ are assigned to the asymmetric stretching mode of the methyl group CH_3_ [51]. According to Arredondo et al. [56], the band at about 1234 and 1430 cm^−1^ corresponds to alcohol C–OH groups. The peak at about 1325 cm^−1^ corresponds to O–H stretching and at 1086 cm^−1^ corresponding to the stretching of C–O [56], the band at about 840 cm^−1^ correspondings to the expansion of the C–O group [51].

FTIR spectrum of Ge/PVA scaffold exhibits characteristic bands of PVA and gelatin. However, some changes are noticeable. For example, the change in the O–H band at 1325 cm^−1^ in the PVA scaffold shifted to 1335 cm^−1^ at the Ge /PVA scaffolds, and 1430 cm^−1^ band of the C–OH group (wide and intense) shifted to 1445 cm^−1^. The gelatin scaffolds bands at 1650 and 1542 cm^−1^ shifted at 1655 and 1551 cm^−1^, respectively. These displacements could be due to hydrogen bonds interactions between C=O groups from gelatin with OH groups in PVA structure.

As in the previously mentioned mixture, the Chi/Ge mixture presented the characteristic bands of both polymers, and there were slight frequency changes in some bands. For example, the band corresponding to the C=O group in chitosan found at 1652 cm^−1^ and in gelatin at 1650 cm^−1^ in the Chi/Ge mixture, which occurs at a lower frequency at 1643 cm^−1^ [57].

These results were similar to those obtained by Chang et al., who suggested that this was because the functional groups of chitosan interacted with the carboxyl groups of gelatin [58]. Finally, in the scaffold composed of Chi/PVA, there was a slight displacement of the band corresponding to the methyl group of PVA at 2944–2911 cm^−1^ [57]. The lack of new peaks in these scaffolds compared to the pure polymer scaffolds indicates that chitosan is compatible with the PVA.

**Figure 7:**
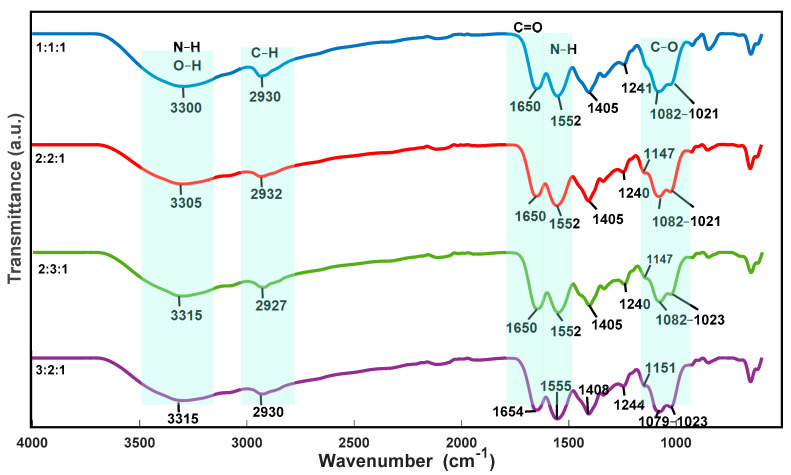
FTIR spectra of ternary blend scaffolds.

The functional groups present in the ternary blends were analyzed by FTIR. The spectra of controls are shown in Figure 7. It exhibits two characteristic bands at 3300–3315 cm^−1^ and 1650–1654 cm^−1^. According to Fan et al. [35] and Ghaderi et al. [51], the three polymers interact through the hydrogen bonding between the amino and hydroxyl moieties.

### 3.4. Swelling Capacity

**Figure 8:**
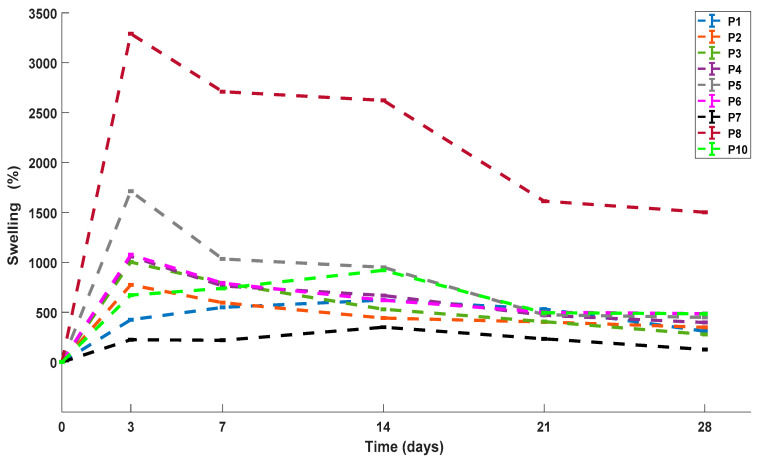
Swelling percentages of ternary blend scaffolds and controls.

Table 2 shows the labels and color codes of all scaffolds for better compression. As an ssential parameter for scaffolds, fluid uptake was studied in phosphate-buffered saline solution (PBS) as the swelling medium. The sample P9 (gelatin scaffold) completely disintegrated after 38 h in the medium. For these reasons, it was not evaluated. This could be explained since gelatin has a good water absorption capacity from five to 10 times greater than its own weight. When gelatin only scaffolds are manufactured, they are fragile, have less flexibility, and their degradation rate is very fragile. Figure 8, it is presented the swelling percentage (SP) of the samples. The ternary blend scaffolds P3 and P4 had a higher swelling percentage SP > 1000%. This is due to the increase in the Chi/Ge ratio, which may be attributed to the presence of polar peptides in gelatin and the excellent water uptake capacity of the chitosan. That could make the scaffold structure loosen, and the macromolecular chains of the system can be extended more easily. These results agree with those reported by Fan et al. and Ghaderi et al. [35,59,60,61]. 

The control Ge/PVA scaffold displayed the SP < 350%. According to Ghaderi et al., “*this is due to probably due to the formation of intra/inter-molecular interactions through hydrogen bonding between hydroxyl groups of C–H and C=O groups of the remaining vinyl acetate units in the PVA backbone*” [51]. The SP (>3000%) of the P8 sample was significantly different from the other samples (*p* < 0.05). In general, all the scaffolds showed good swelling capacity, which is typical of systems of this type given their high porosity, the characteristics mentioned above of polymers, and affinity for water. This behavior is advantageous for cell culture since these are immersed in an aqueous medium. The scaffold must have the ability to allow the absorption of nutrients from and to the outside of the scaffold.

The swelling capacity of the scaffolds is an essential requirement for them to be used in the three-dimensional (3D) culture. This ability to absorb fluids allows nutrients and cellular metabolites to diffuse into the scaffolds, which positively influences the maintenance of cultured cells [62]. Therefore, the swelling capacity is decisive to know its stability in the culture medium [51] and in the chemical and physical characteristics of the scaffolds before and after cell seeding [63]. For example, the binding between cells and scaffold is mediated and controlled by the ability of adhesion proteins to absorb onto the surface of the scaffold so that cells will not be able to adhere to the surface if cell adhesion proteins are not present. Consequently, it has been shown that maximum cell adhesion to scaffold surfaces occurs on surfaces of moderate wettability. As high or low wettability surfaces discourage cell adhesion [64].

### 3.5. Degradation

**Figure 9:**
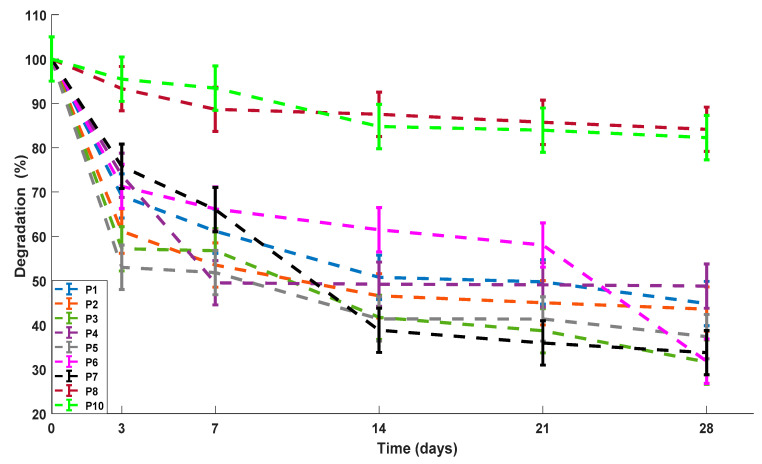
Percentage of degradation for the ternary blend scaffolds and controls.

On day zero, all the scaffolds start with their mass at 100%, as shown in the Y-axis of Figure 9. This figure shows that the percentage of degradation (DP) of the scaffolds increases as time goes by. This behavior is the same in all samples. Although, on day 28 some samples degraded more than others. For example, the ternary blend P3 had a higher percentage of degradation on day 28 compared to the other ternary blends. This could be beause it is the one with the highest weight ratio of gelatin. On the other hand, binary blends had a higher DP compared to ternary blends. Finally, it was observed that controls P8 and P10 have a slow degradation, where at day 28, their DP is less than 20%. In the individual and multiple comparisons, significant differences (*p* < 0.05) were found in the percentage of degradation of the P8 and P10 controls compared to the other samples, which is evident in Figure 9 and could be due to the slow degradation in vitro both chitosan (P8) and PVA (P10) [65].

The disintegration speed of the scaffolds is essential because if they disintegrate very quickly, as the P9 scaffold did, they do not allow the β cells to adhere, divide and proliferate. Ideally, the β cells deposit the ECM as the scaffolds degrade to restore tissue function [27].

### 3.6. Compressive Behaviors of the Scaffolds

**Figure 10:**
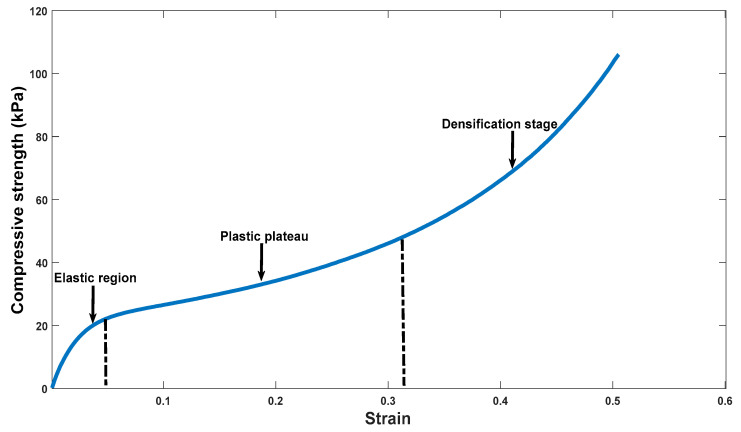
Example of the compressive strength-strain curve that occurs in scaffolds.

The compressive strength-strain curve of all the samples presented three characteristic zones of this type of material. To observe and explain these three zones, the compressive strength-strain curve of the ternary mixture P3 is taken as an example, as observed in Figure 10. In this figure, the three zones can be observed: elastic zone, plastic zone, and a third zone been named as a densification state according to the authors Jiang et al., and Matinfar et al. This state of densification occurs in these materials, since with the compression force, the pores collapse, reducing the volume of porosity, allowing the material to have more resistance [41,62].

**Figure 11:**
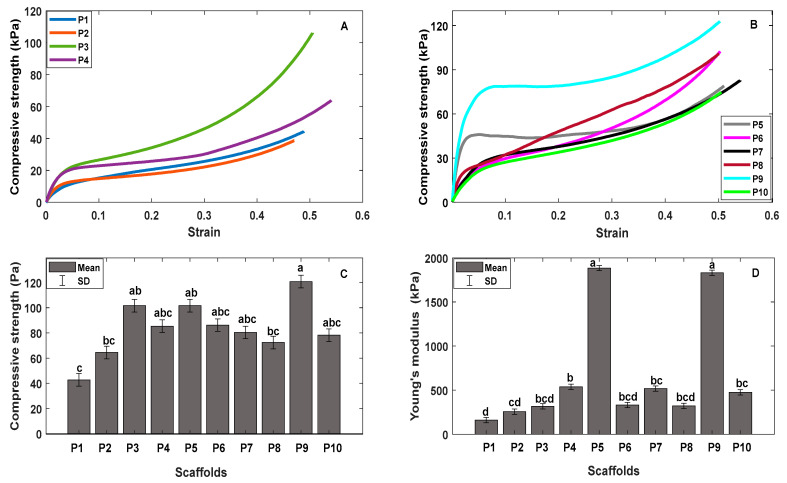
(**A**) Compressive strength-strain curve for ternary blend scaffolds. (**B**) Compressive strength-strain curve for controls scaffolds. (**C**) Compressive strength of scaffolds. (**D**) Compressive modulus of scaffolds. The data are presented as mean ± SD; the different letters indicate significant compressive strength and compressive modulus differences (*p* < 0.05).

Elastic zone, plastic zone, and densification stage were observed in ternary blends Figure 11A and all controls as seen in Figure 11B. It was observed that the addition of gelatin increased the rigidity and compressive strength in the samples, as seen in Figure 11C. For example, the compressive strength of P8 (chitosan) was at 72.4 Pa when gelatin P5 (Chi/Ge) was added, and this modulus increased to 101.6 Pa. Furthermore, P3 has higher compressive strength, 101.6 Pa followed by the P4 scaffold with a compressive strength of 85.35 Pa; since these samples have the highest weight ratio of gelatin compared to the ternary mixtures P1 and P2. Significant differences were found in the compressive strength in the ternary blend P1 and the control P9 with respect to the other samples (*p* < 0.05), in the multiple comparisons using the Tukey method, significant differences (*p* < 0.05) were observed in the compressive strength of the ternary blend P1 with respect to the control P5, P9, and ternary blend P3.

In Figure 11D, we have Young’s modulus of ternary blends and controls. Here we observe that gelatin also has the greatest rigidity and that the addition of gelatin increases Young’s modulus of both controls and ternary blends. As can be seen, the P8 scaffold has Young’s modulus of 321.45 kPa, and when gelatin P5 was added, this modulus increased to 1884.72 kPa. Furthermore, something similar happened with the P10 sample (PVA), which had Young’s modulus of 476 kPa, and with the addition of gelatin P7 (Ge/PVA), the modulus increased to 518 kPa. The ternary blend P4 had a higher Young’s modulus 537.1 kPa, followed by the ternary blend P3 317.2 kPa. Significant differences were found in Young’s modulus of ternary blend P1and P2; and in the controls P5, and P9. When making multiple comparisons, significant differences (*p* < 0.05) were observed in Young’s modulus of in ternary blends P3 and P4 with respect to samples P5 and P9.

An essential requirement that scaffolds must meet is that they allow cell culture, providing an environment similar to the native one, so that the cells synthesize new tissue in the scaffold and that this is capable of delivering sufficient mechanical support to resist the physiological loads in the scaffold implementation in situ [66]. This is very important since the native environment of the cells has a unique microstructure and compositions of the ECM, as well as biomechanics that maintains the different signals for the cells that reside there, allowing the cells to differentiate, migrate and proliferate [9,17,67]. These forces have been shown to affect not only biology but also cellular function and underlying mechanisms. The study published by Mamidi et al. transcriptional changes directly affect in vivo and in vitro the cells differentiation and intracellular cytoskeletal dynamics, those changes are due the extracellular environment [68,69]. Even mechanical resistance is necessary for scaffold pretreatments before cell culture [62].

### 3.7. Cytocompatibility of Ternary Blend Scaffolds and Controls

**Figure 12:**
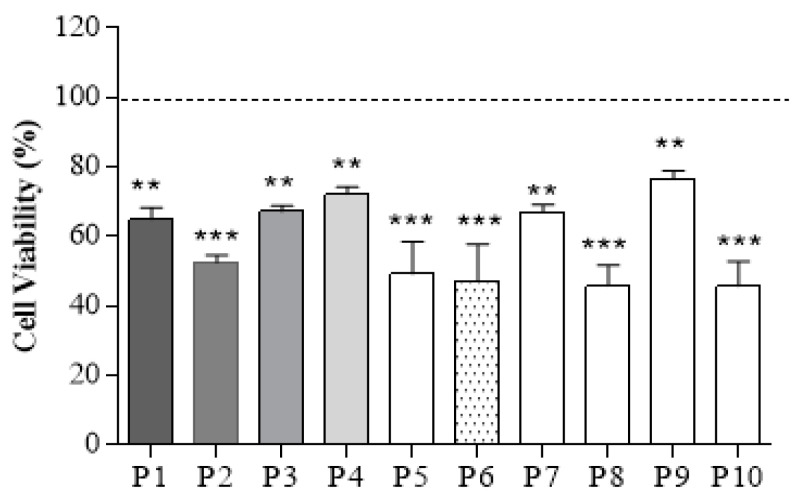
Cell viability of beta pancreatic cells. MTT assay results showing the percentage of viable cells on P1 to P10 samples after a culture period of 48 h. “**, ***” denotes a statistically significant difference (*p* < 0.05) in cell number concerning the tissue culture plastic (TCP). Data points represent mean ± SD (*n* = 3).

To evaluate if there is a cytotoxic response in the gelatin, chitosan, and PVA scaffolds, the percentage of cell viability was evaluated. As shown Figure 12, all the samples showed cell viability greater than 50% regardless of the polymer proportion. Although sample P9 (gelatin) disintegrated in the culture medium, it was the one with the highest viability; This could be explained since gelatin, being derived from collagen, shares similarities with it in terms of its molecular structure and function [70]. 

On the other hand, ternary mixtures P1 and P2 have lower cell viability since they have a higher proportion of PVA; and PVA does not favor the porosity and interconnectivity in the scaffold. For example, P2 has a higher Chi/PVA weight ratio than P1, which could explain why it has the lowest cell viability.

Finally, the ternary mixtures P3 and P4 maintained their structure during the cultivation time, thanks to their mechanical properties. As seen in Figure 11C,D, the addition of gelatin improved the compressive strength and stiffness of these blends. But, the pure polymer scaffold (P9). However, it has greater viability, and cannot offer that structural support to cells due to its rapid degradation in vitro due to the presence of polar peptides that compose it [51]. Additionally, P3 and P4 presented the better size of pore and porosity percentage, which could explain the better cell viability, higher to 65 and 70%, respectively. These results suggest that these ternary mixtures have mild toxicity according to ISO 10993-5: 2009 for BRIN-BD11 cells [71].

In the results of cell viability, it was observed that the percentage of porosity, the pore size, the mechanical properties, the degradation rate, and the proportion of polymer are fundamental in the scaffold since these factors can favor or disfavor the viability of the cells BRIN-BD11 in culture. It was observed that the addition of gelatin in the ternary blends P3 and P4 favored cell viability since these scaffolds provided cell-binding sites and a 3D microenvironment that favored the viability of these cells, which is characteristic of scaffolds containing gelatin [70]. On the other hand, it was observed that the PVA (P10) scaffold had low cell viability, which is due to the difficulty of cells to adhere to the scaffold due to the highly hydrophilic nature of PVA [64,72], so it could be observed that the PVA content in ternary blend had a direct effect on cell viability.

Assessing cell viability is essential for this scaffold since its main objective is to support β cells. Therefore, the scaffolds cannot be cytotoxic, provoke an inflammatory or chronic response since it could affect cell adhesion. This is crucial since various cellular functions depend on it, such as growth, differentiation, migration, cell survival, and revascularization of the pancreatic islet [9,27,73,74].

## 4. Conclusions

The pre-freezing temperature in the freeze-thaw cycles, the freeze-drying, and the Chi/Ge content favored the formation of pores in the scaffolds. Therefore, this pore formation was better in the scaffolds of ternary blends (P1-P4), which have a heterogeneous and random porosity, resulting in scaffolds with porosity greater than 80%.

The high porosity of scaffolds was found to influence mechanical properties. These properties are essential because scaffolds must have the ability to resist loads and transfer them correctly. The addition of gelatin to the scaffolds is believed to significantly improve compressive strength and Young’s modulus due to forming a polyelectrolyte complex between the polymers. In addition, these mechanical properties allow the scaffolds to be easily manipulated when being implemented in the crop. These could maintain rigidity while allowing the deposition of the ECM.

The three polymers used had different effects on the scaffolds. Gelatin improved mechanical properties and cell viability but decreased the percentage of scaffold degradation. The Chi/Ge ratio increased the percent swelling, the pore size, and the porosity. On the other hand, the Chi/PVA ratio improved the degradation rate but decreased cell viability.

Most of the ternary blens scaffolds showed good pore size, porosity, water absorption capacity, and degradation percentage characteristics, but, it was found that the ternary mixtures P3 and P4 presented the best characteristics to be used in 3D cultures due to their biological, physical-chemical, and mechanical properties.

Ternary blends showed a controllable degradation rate compared to binary blends. They preserved their porous structure after 28 days of being submerged in PBS, indicating that these scaffolds have good structural stability, allowing the passage of nutrients to the outside of the scaffold and serving as structural support to beta cells during the culture time.

Ternary blend scaffolds were shown to have highly porous and interconnected structures with different types of pores. The ternary blends presented a porosity percentage higher than 80%, unlike the controls that gave a lower porosity percentage. Finally, ternary blend scaffolds have the most controllable swelling rate than pure polymer scaffolds; This makes it possible to know its fluid adsorption capacity over time to obtain its prior stabilization in the culture medium. Ternary blends P3 and P4 showed mild toxicity according to ISO standard for BRIN-BD11 cells. These data indicate that chitosan, gelatin, and PVA scaffolds show promise for β-cell culture.

## Figures and Tables

**Table 1:** Compositions and ID of Chi/Ge/PVA ternary blend scaffold and controls.

Scaffolds	Blending Ratio	ID	Color
Ternary blend scaffolds	1:1:1	P1	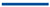
	2:2:1	P2	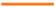
	2:3:1	P3	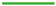
	3:2:1	P4	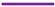
Control	1:1:0	P5	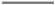
	1:0:1	P6	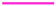
	0:1:1	P7	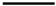
	1:0:0	P8	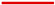
	0:1:0	P9	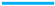
	0:0:1	P10	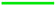

**Table 2:** Porosities of Samples scaffolds.

Scaffolds	Porosity (%)
P1	80 ± 0 ^a,b,c^
P2	90 ± 2.7 ^a^
P3	85.71 ± 0 ^a,b^
P4	94.06 ± 0.7 ^a^
P5	87.85 ± 6.1 ^a^
P6	83.33 ± 2.4 ^a,b^
P7	55.56 ± 9.6 ^c^
P8	91.40 ± 1.5 ^a^
P9	73.89 ± 6.7 ^a,b,c^
P10	58.73 ± 13.8 ^b,c^

Note: the data is presented as mean ± standard deviation; different letters indicate significant differences with a significance level of 99.9%. In these results, the table shows that group A contains samples P1–P6 and P8 and P9, group B contains samples P1, P3, P6 and P9, and P10, and group C contains samples P1, P7, P9, and P10. Samples P1 and P9 are in all three groups. The differences between the means that share a letter are not statistically significant.

## Data Availability

The data presented in this study are available on request from the corresponding author.
